# Effects of a cafeteria-based sustainable diet intervention on the adherence to the EAT-Lancet planetary health diet and greenhouse gas emissions of consumers: a quasi-experimental study at a large German hospital

**DOI:** 10.1186/s12937-024-00981-x

**Published:** 2024-07-18

**Authors:** Laura Harrison, Alina Herrmann, Claudia Quitmann, Gabriele Stieglbauer, Christin Zeitz, Bernd Franke, Ina Danquah

**Affiliations:** 1 Heidelberg Institute of Global Health, Medical Faculty and University Hospital Heidelberg, Heidelberg University, Im Neuenheimer Feld 130.3, Heidelberg, 69120 Germany; 2 Ifeu - Institut Für Energie- Und Umweltforschung Heidelberg gGmbH, Wilckensstr. 3, Heidelberg, 69120 Germany; 3 Hertz-Chair Innovation for Planetary Health, Center for Development Research (ZEF), University of Bonn, Genscherallee 3, Bonn, 53113 Germany

**Keywords:** Difference-in-differences, Plant-based diets, Greenhouse gas emissions, EAT-Lancet Planetary Health Diet, Vegan

## Abstract

**Background:**

Sustainable diets contribute to improving human health and reducing food-related greenhouse gas emissions (GHGE). Here, we established the effects of a facility-based sustainable diet intervention on the adherence to the EAT-Lancet Planetary Health Diet and GHGE of consumers.

**Methods:**

In this quasi-experiment, vegan menus and educational material on sustainable diets were provided in the largest cafeteria of a German hospital for 3 months. Regular customers (> 1/week) in this cafeteria (intervention group) and in all other hospital cafeterias (control group) completed a questionnaire about their sociodemographic and dietary characteristics before and after the intervention period. We calculated difference-in-differences (DID), their 95% confidence intervals (CIs), and *p*-values for the adherence to the EAT-Lancet Planetary Health Diet Index (PHDI; 0–42 score points) and food-related GHGE. The protocol was registered at the German Clinical Trial Register (reference: DRKS00032620).

**Findings:**

In this study population (*N* = 190; age range: 18–79 years; women: 67%; highest level of formal education: 63%), the mean baseline PHDI (25·1 ± 4·8 vs. 24·7 ± 5·8 points) and the mean baseline GHGE (3·3 ± 0·8 vs. 3·3 ± 0·7 kg CO2-eq./d) were similar between the intervention (*n* = 92) and the control group (*n* = 98). The PHDI increase was 0·6 points (95% CI: -0·4, + 1·6) higher in the intervention group than in the control group. This trend was stronger among frequent consumers of the vegan menu than among rare and never consumers. No between-group difference was seen for GHGE changes (DID: 0·0; 95% CI: -0·2, + 0·1 kg CO2-eq./d).

**Interpretation:**

Pending verification in a longer-term project and a larger sample, this quasi-experiment in a big hospital in Germany suggests that offering vegan menus and information material in the cafeteria enhances the adherence to healthy and environmentally friendly diets among regular customers. These findings argue for making sustainable food choices the default option and for improving nutrition literacy.

**Funding:**

Federal Ministry of Economic Affairs and Climate Action (BMWK), Else-Kröner-Fresenius Foundation (EKFS), Robert-Bosch Foundation (RBS).

**Supplementary Information:**

The online version contains supplementary material available at 10.1186/s12937-024-00981-x.

## Background

“We are on the edge of an abyss — and moving in the wrong direction. […] The world must wake up. […] The problems we have created are problems we can solve.”, as stated by Antonio Guterres, secretary-general of the United Nations [1]. Climate change is the largest threat to human health in the twenty-first century [2, 3]. To mitigate climate change and its adverse effects, a reduction of greenhouse gas emissions (GHGE) to the largest possible extent in every sector is needed [3]. Today, the food sector is responsible for one-third of global GHGE [4].

Diets low in animal products, have an enormous potential to reduce food-related GHGE by up to 70% [5]. In addition, plant-based diets, in comparison to diets high in animal-products, contribute to the prevention of cardiometabolic diseases, improve the risk of cancers, and reduce overall premature mortality [6]. Sustainable diets are defined as “diets with low environmental impacts that contribute to food and nutrition security, and to healthy lives for present and future generations” [7]. To provide “a safe operating space” for sustainable diets, the EAT-Lancet Commission developed the Planetary Health Diet (PHD) in 2018. This reference diet could feed up to 10 billion people in 2050 while remaining inside planetary boundaries. The PHD is plant-based and recommends moderate intakes of poultry and seafood, processed meat, red meat, added sugar, and refined grains [6].

Multiple strategies can contribute to implementing this dietary shift on a global scale, including policies to support the adoption of sustainable diets, raising public awareness on the environmental, health and ethical benefits of these diets, and increasing accessibility and affordability [8]. While dietary interventions at the individual level rapidly show improvements in cardiometabolic parameters, these effects are often not sustained in the long term [9]. Previously, facility-based dietary interventions produced long-term dietary changes through modifications of the food environment [10]. These modifications are especially effective when components of models for behaviour change, e.g., the transtheoretical model (TTM) and the COM-B model, are adopted [11, 12]. The TTM describes different stages of behaviour change, while the COM-B model suggests increasing capability (C), opportunity (O), and motivation (M) for dietary changes. Indeed, knowledge on sustainable diets is lacking in the European population [13], despite its beneficial influence on pro-environmental behaviour [14]. Educational dietary interventions have improved adherence to promoted diets [15]. Education is useful at any stage of the TTM and provides the basis for goal-directed behavioural change. Approximately half of the hospital employees’ waking hours are spent at work [16]. Therefore, consistent exposure to changes in the work-related food environment may help navigate individuals through the different stages of change and achieve sustainable dietary habits.

Currently, the majority of the general population in Germany and hospital food suppliers barely comply with the PHD recommendations [17]. Although previous research provides useful guidance on facility-based dietary interventions, [18] it is largely unknown to what extent plant-based meals at the facility level can improve the adherence to the PHD. Indeed, sustainable diets may clash with culinary traditions and have become an ideological, if not even philosophical, topic [19]. Therefore, empirical evidence on effective sustainable diet interventions are urgently needed [16].

Our study aimed to establish the effects of a cafeteria-based intervention with vegan menu offering and nutrition information in a German hospital on the customers’ adherence to the PHD and food-related GHGE.

## Methods

### Study design

We used a quasi-experimental pre and post study design with an intervention group and a control group. This project is part of the “Climate Change Mitigation through Optimizing Supply Chains in Hospitals” (KliOL) project with the goal of reducing the greenhouse gas emissions from hospital supply chains by 7%.

### Intervention

We conducted this study at Heidelberg University Hospital in Germany, which has 7 cafeterias for employees. The standard lunch menu, offered to patients and employees, comprised two meat-and-fish menu lines (75%) and one vegetarian menu line (25% of orders) each day.

The intervention programme aimed to increase the sustainability of consumers’ dietary behaviour, and targeted consumers at different stages of the TTM [11], using all components of the COM-B model of behaviour change. The intervention was co-designed by kitchen staff, nutritionists, corporate health management staff and researchers from medicine, nutrition science, and epidemiology. The intervention programme comprised two components: (1) daily substitution of one meat-and-fish-menu line with a vegan menu line, and (2) one-time provision of printed information material on the importance of dietary practices for climate change and health, including a link to vegan online-recipes. For more information, see Supplementary text 1). The intervention period lasted three months from mid-January to mid-April 2023.

### Allocation of the intervention

The intervention programme was allocated at the facility level. We introduced our intervention in one hospital cafeteria (= intervention cafeteria). In all other cafeterias, the standard hospital meals were maintained (= control cafeterias). Due to the systemic nature of this intervention, allocation concealment and blinding were not possible.

### Participants

Regular consumers of cafeteria meals (≥ once/week) at follow-up, mostly hospital employees, were eligible for participation. Inclusion criteria were age ≥ 18 years at baseline and not following a medically prescribed dietary regimen. We asked participants to state their gender identity rather than sex, as gender and traditional gender role conformity play a role in the openness towards adopting more sustainable diet patterns [20]. We recruited participants in front of hospital cafeterias and via institutional e-mailing lists. All participants provided written informed consent.

### Data collection

We conducted questionnaire-based surveys at baseline and at endline. The questionnaires were completed online or as paper-and-pencil versions. We assessed demographic and socioeconomic characteristics (Table 1), self-reported hours of moderate-intensity physical activity, and self-assigned type of dietary practice (vegan, vegetarian, pescatarian, flexitarian, mixed diet) (Supplementary Text 2). We administered process evaluation questions, e.g., on the frequency of the vegan menu consumption and the use of the information material. To minimize the time expenditure for our study participants, usual dietary intake over the past 3 months was assessed by means of a semi-quantitative food frequency questionnaire (FFQ) with 116 food items. The FFQ was developed by the study team based on the German food-based dietary guidelines to include more plant-based food items and is described in more detail in Supplementary text 3.

### Outcomes

This paper is part of a larger study (DRKS00032620) with the primary outcome mental wellbeing. The present paper reports on two secondary outcomes: (1) the Planetary Health Diet Index (PHDI) and (2) individual food-related GHGE of consumers’ diets.

We employed the PHDI constructed by Stubbendorff et al., which measures adherence to the EAT-Lancet PHD on a score from 0 (= least) to 42 points (= most sustainable diet) [21]. Recommended food groups (*n* = 7) received higher points and unrecommended food groups (*n* = 7) received lower points for more frequent consumption, respectively [21]. GHGE were calculated in kg CO_2_eq./day using factors from life-cycle analyses from cradle-to-shelf, including land use change, for each food item provided by the ifeu [22]. For more details, see Supplementary Table 1 and Supplementary Text 3.

### Sample size

The sample size calculation (Supplementary Text 4) was performed for the primary outcome mental wellbeing (Warwick-Edinburgh-Mental-Wellbeing-Scale, WEMWBS) [23]. For previously reported effect sizes of 0.18 to 0.30 WEMWBS points, the estimated sample size was 90 to 245 participants. Sample size calculations were not performed for the secondary outcomes.

### Statistical methods

#### Missing data handling

Participants with implausible energy intake or missing data on more than five food items were excluded from the analysis. All other missing food items were substituted as described in Supplementary Text 5. For GHGE analyses, we removed all outliers (> 1·5 interquartile range) for pre-post-differences in GHGE. We assumed that such outliers stem from implausible intake changes in food groups with a high environmental footprint, such as beef.

#### Descriptive statistics

Demographic, socioeconomic, and lifestyle characteristics were compared between groups at baseline and follow-up, using Chi-square tests. We also calculated standardized mean differences of the baseline characteristics between groups. In addition, we examined whether covariates with significant differences in groups or over time qualified as potential confounders and calculated their time-varying effects on the outcomes in the control group [24].

We explored the distributions of the PHDI and food-related GHGE in box-plots by categories of the self-assigned dietary types and by the number of cafeteria meals consumed per week. Data are shown by groups and for baseline and follow-up examinations. Results of the process evaluation are presented for the intervention group only. To show the relationships between intervention uptake and outcome variables, we calculated mean PHDI and mean GHGE by the intake frequency of the vegan menu.

#### Difference-in-differences (DID) analysis

The intervention effects were calculated using difference-in-differences (DID) analyses. The participants were categorised into the intervention or the control group, depending on their most frequented cafeteria at follow-up.

DID analysis estimates the difference between the changes in the outcome measures in the intervention and in the control group over time. The DID analysis assumes that the outcomes of both groups would follow “parallel trends” over time in the absence of the intervention [25].

We performed linear regression analyses to derive the DID estimates, their 95% confidence intervals (CIs), and *p*-values using SAS 15·1. We fitted three models: Model 1 was unadjusted, Model 2 was adjusted for educational attainment, and Model 3 was adjusted for all covariates (see Table 1) [24, 26].

Finally, we conducted exploratory DID analyses for single food groups to identify the main contributors to the observed changes in PHDI and GHGE. These analyses were performed for points per PHDI category, for grams per PHDI category calculated as detailed in Supplementary Table 1, and for GHGE measured in kg CO_2_eq, based on the food groups categorized in the questionnaire. We also stratified the DID analyses for both outcomes by gender.

#### Sensitivity analyses

As a sensitivity analysis for the fully adjusted Model 3 and to account for differences in baseline characteristics, we additionally employed propensity score weighting (describing the conditional probability of being assigned to groups, Model 4). Propensity scores are frequently used in combination with DID analysis to minimize case mix differences.[27] Due to the possibility of switching groups, we performed an additional intention-to-treat analysis (ITT) and calculated the local average treatment effect (LATE). The LATE estimate applies only to the subgroup that is compliant when assigned to the intervention [26]. Additionally, we performed a random split of the sample and randomization inference to ascertain the probability of finding larger effects under all possible random assignments of participants to groups using STATA MP18 [28].

### Role of the funding source

The funders had no role in the study design, data collection, data analysis, data interpretation, writing of the manuscript or any aspect pertinent to the study. None of the authors were paid to write this article by a pharmaceutical company or other agency. Authors were not precluded from accessing data in the study, and they accept responsibility for submission for publication.

## Results

### Recruitment and follow-up

**Fig. 1:**
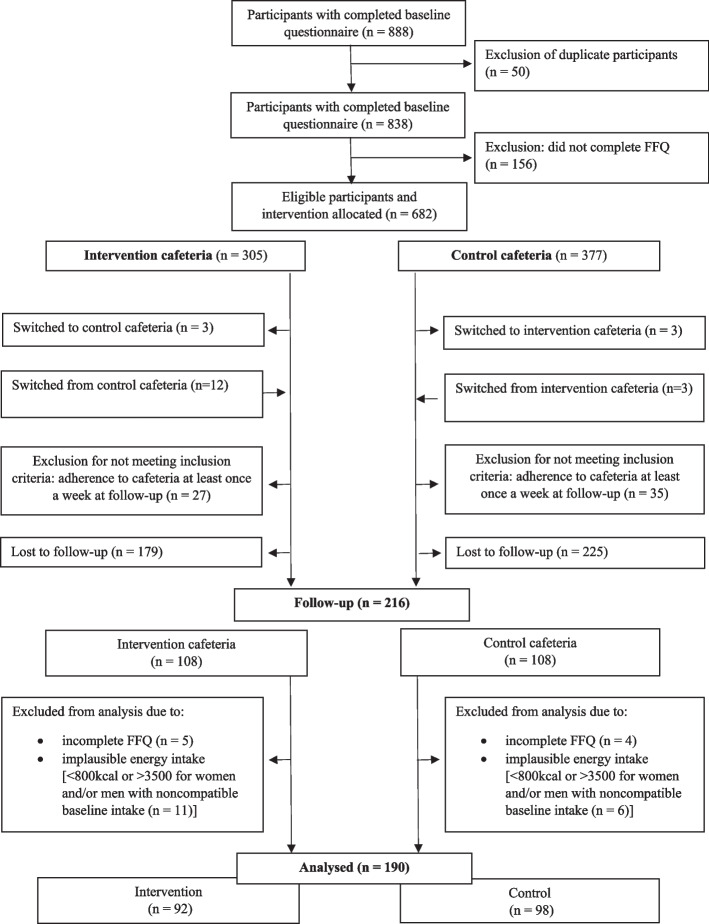
Flow chart of final sample

Questionnaires were completed at baseline (07 December 2022 – 15 January 2023) and at follow-up (27 March 2023 – 30 April 2023). Out of the 682 participants who completed the FFQ at baseline, 190 participants were analysed (control: *n* = 98, intervention: *n* = 92). Fig. 1 shows the number of participants who completed the baseline and follow-up surveys, by group.

### Baseline characteristics

The baseline characteristics for each group are presented in Table 1. There were no differences in age, gender, marital status, monthly income, number of children in the household, age of the youngest child, and physical activity. However, the intervention group less frequently attained high educational degrees (61% vs 64%) and more frequently consisted of students (10% vs 1%). The same distributions were observed for the standardized mean differences at baseline (Supplementary Fig. 1). We identified education as a potential confounder because of its time-varying effects on the PHDI [24].

**Table 1:** Characteristics of 190 hospital employees at baseline and follow-up by intervention group

**Characteristics**	**Baseline**			**Follow-up**		
	**Control**	**Intervention**	**p**	**Control**	**Intervention**	**p**
n	98	92		98	92	
**Age (years)**			0·19			0·26
18–29	12 (12·24%)	20 (21·74%)		11 (11·22%)	19 (20·65%)	
30–39	31 (31·63%)	24 (26·09%)		32 (32·65%)	24 (26·09%)	
40–49	25 (25·51%)	14 (15·22%)		24 (24·49%)	15 (16·30%)	
50–59	21 (21·43%)	25 (27·17%)		22 (22·45%)	24 (26·09%)	
60–79	9 (9·18%)	9 (9·78%)		9 (9·18%)	10 (10·87%)	
**Gender**			0·25			0·46
Male	35 (36·08%)	26 (28·26%)		33 (34·38%)	27 (29·35%)	
Female	62 (63·92%)	66 (71·74%)		63 (65·63%)	65 (70·65%)	
Diverse *	1			2		
**Marital status**			0·49			0·14
Single or short-term relationship	34 (34·69%)	38 (41·76%)		33 (33·67%)	43 (46·74%)	
Single after long relationship	3 (3·06%)	4 (4·40%)		3 (3·06%)	4 (4·35%)	
In a long-term relationship	61 (62·24%)	49 (53·85%)		62 (63·27%)	45 (48·91%)	
Missing		1				
**Monthly income per household (€)**			0·81			0·13
0 < 20,000	4 (4·08%)	7 (7·61%)		2 (2·11%)	6 (6·67%)	
20,000 < 60,000	29 (29·59%)	29 (31·52%)		36 (37·89%)	32 (35·56%)	
60,000 < 100,000	28 (28·57%)	24 (26·09%)		20 (21·05%)	26 (28·89%)	
≥ 100,000	21 (21·43%)	16 (17·39%)		27 (28·42%)	14 (15·56%)	
"No answer “	16 (16·33%)	16 (17·39%)		10 (10·53%)	12 (13·33%)	
Missing				3	2	
**Educational degree**			0·02			0·33
High: Higher education or higher vocational training	63 (64·29%)	56 (60·87%)		61 (62·24%)	56 (60·87%)	
Medium: A levels or completed vocational training	26 (26·53%)	35 (38·04%)		28 (28·57%)	32 (34·78%)	
Low: Secondary School/GCSE without vocational training	9 (9·18%)	1 (1·09%)		9 (9·18%)	4 (4·35%)	
**Number of children in household**			0·08			0·09
0	60 (61·22%)	70 (76·09%)		58 (59·1%8)	68 (73·91%)	
1	16 (16·33%)	8 (8·70%)		19 (19·39%)	10 (10·87%)	
≥ 2	22 (22·45%)	14 (15·22%)		21 (21·43%)	14 (15·22%)	
**Age of youngest child (years)**			0·29			0·26
no children	48 (48·98%)	51 (56·04%)		46 (46·94%)	51 (56·04%)	
0 < 10	18 (18·37%)	15 (16·48%)		20 (20·41%)	16 (17·58%)	
10 < 18	16 (16·33%)	6 (6·59%)		13 (13·27%)	5 (5·49%)	
18 < 30	11 (11·22%)	14 (15·38%)		13 (13·27%)	16 (17·58%)	
≥ 30	5 (5·10%)	5 (5·49%)		6 (6·12%)	3 (3·30%)	
missing		1			1	
**Occupation**			0·01			0·04
Student/Voluntary service	1 (1·02%)	9 (9·78%)		2 (2·04%)	8 (8·79%)	
Employee or civil servant	97 (98·98%)	83 (90·22%)		96 (97·96%)	83 (91·21%)	
missing					1	
**Moderate intensity physical activity (h/week) (self-reported)**			0·69			0·21
< 1	25 (25·51%)	19 (20·65%)		23 (23·47%)	13 (14·13%)	
1 < 2·5	34 (34·69%)	39 (42·39%)		34 (34·69%)	44 (47·83%)	
2·5 < 5	27 (27·55%)	25 (27·17%)		30 (30·61%)	27 (29·35%)	
≥ 5	12 (12·24%)	9 (9·78%)		11 (11·22%)	8 (8·70%)	

Data are shown as n (%), *p*-values were calculated by Chi-square test. * Coded as missing for Chi-square test·

When comparing the baseline characteristics of included participants (*n* = 190) with those of individuals who were lost to follow-up (*n* = 492), we found more married participants, more individuals with own children, and fewer individuals aged 18–29 years in the analytical dataset.

### Uptake of intervention components

Thirty percent of all participants and 20% of mixed diets consumers reported choosing the vegan menu at least 3-times a week. Regarding information material, 50% did not see the material, 23% read the flyer, and 11% read the online-recipes. One-third of the consumers of the flexitarian and mixed diets stated that they were motivated by the cafeteria menu to adopt more sustainable dietary practices at home.

### Distributions of the EAT-Lancet planetary health diet index and greenhouse gas emissions

**Fig. 2:**
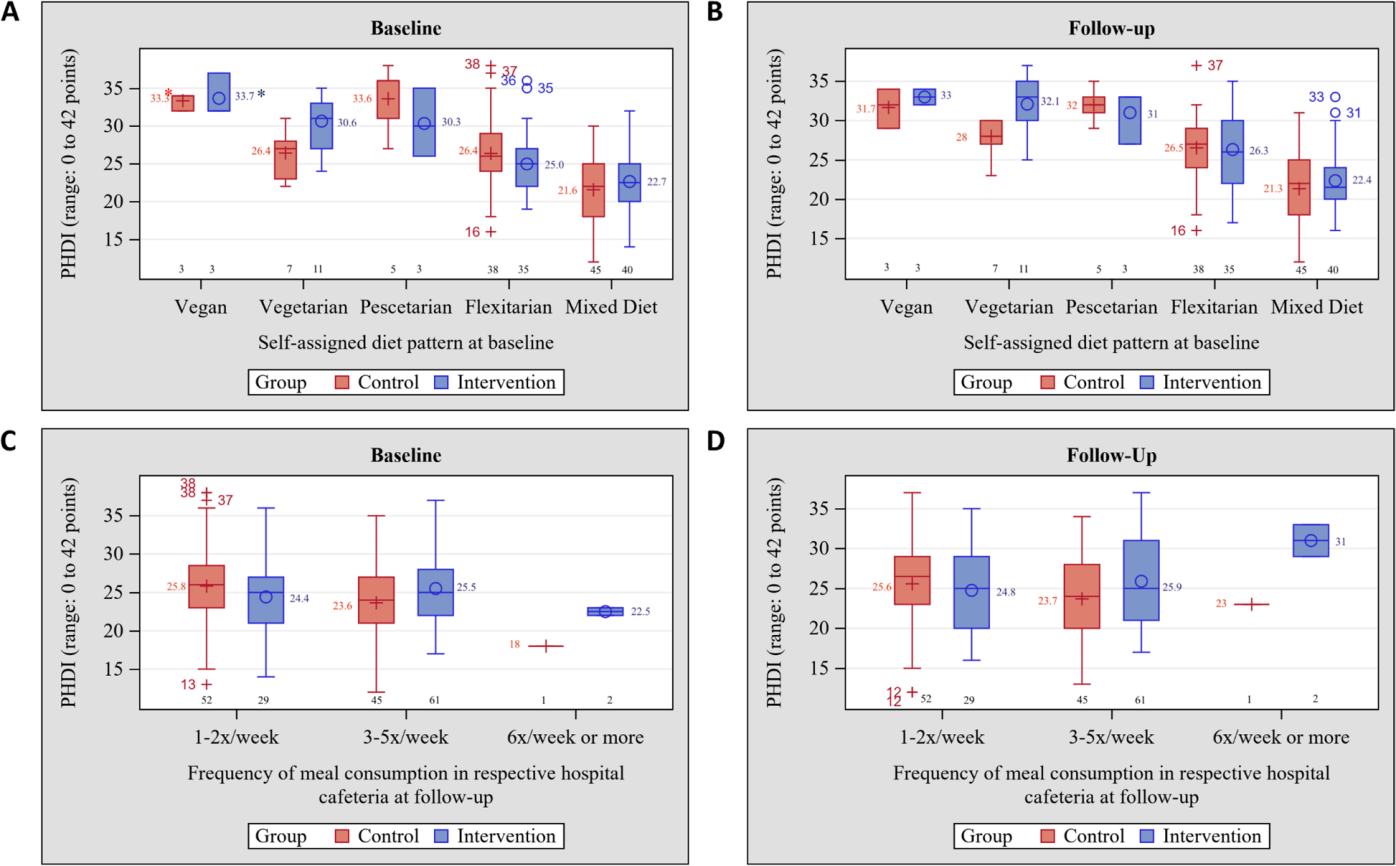
Distributions of the EAT-Lancet Planetary Health Diet Index (PHDI) by intervention groups at baseline and at endline. **mean value, (A) PHDI at baseline according to self-assigned dietary type at baseline, (B) PHDI at follow-up according to self-assigned dietary type at at baseline, (C) PHDI at baseline according to the frequency of consumed cafeteria meals at baseline, (D) PHDI at follow-up according to the frequency of consumed cafeteria meals at baseline

At baseline, mean PHDI was 24·7 ± 5·8 score points in the control group and 25·1 ± 4·8 score points in the intervention group. Fig. 2A shows the distributions of PHDI by intervention group at baseline according to self-assigned dietary types. In both groups, individuals with vegan dietary practice had the highest mean PHDI, followed by pescatarian, vegetarian, flexitarian, and mixed dietary types. At follow-up, PHDI distributions did not change for the vegan, pescatarian, flexitarian and mixed diets, but mean PHDI was increased among individuals on vegetarian diets in the intervention group (Fig. 2B).

**Fig. 3:**
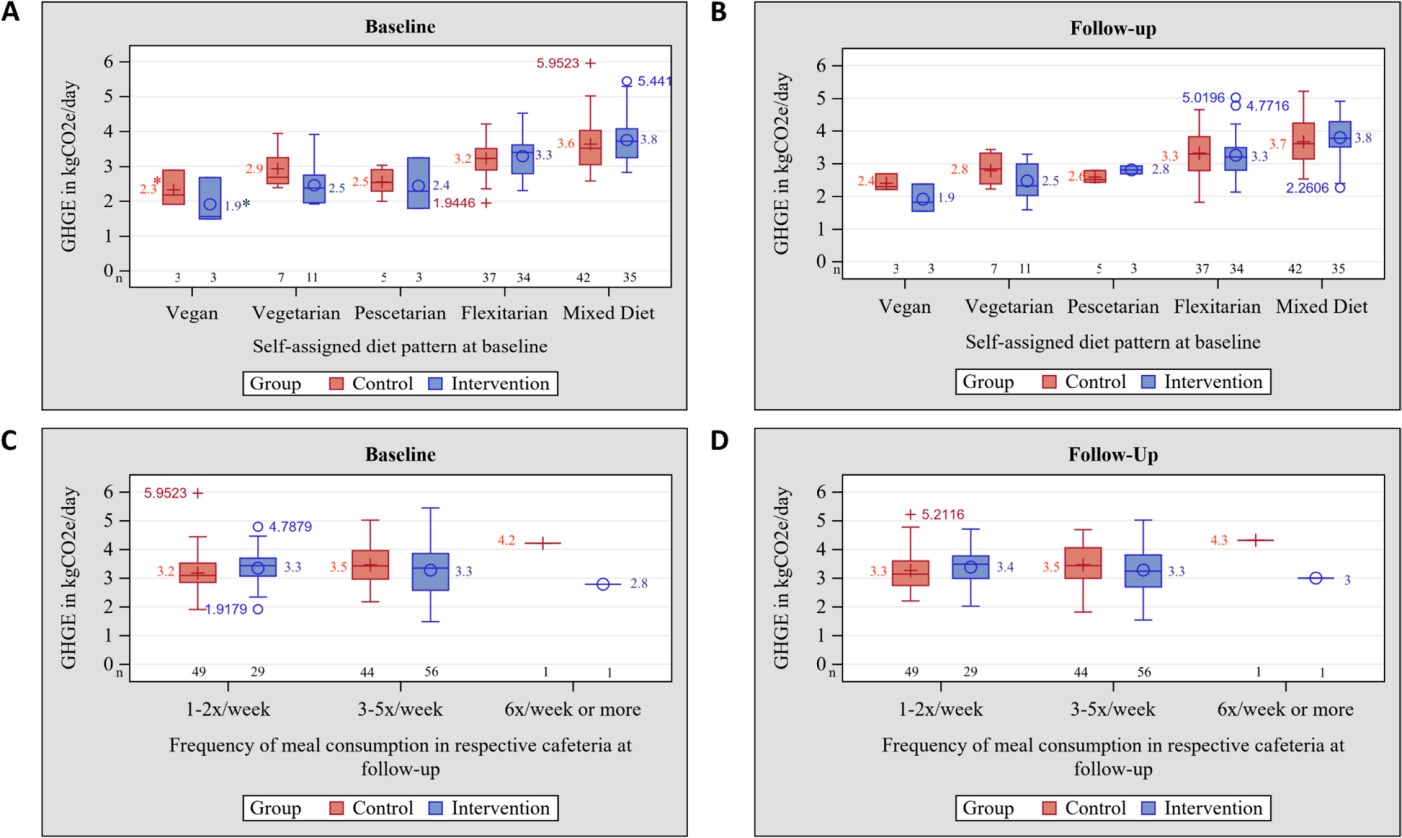
Distributions of greenhouse gas emissions (GHGE) in kg/day by intervention groups at baseline and at endline. **mean value, (A) GHGE at baseline according to self-assigned dietary type at baseline, (B) GHGE at follow-up according to self-assigned dietary type at at baseline, (C) GHGE at baseline according to the frequency of consumed cafeteria meals at baseline, (D) GHGE at follow-up according to the frequency of consumed cafeteria meals at baseline

With regard to GHGE, the mean baseline values were 3·32 ± 0·68 kg CO_2_eq./day in the control group and 3·29 ± 0·81 kg CO_2_eq./day in the intervention group. Fig. 3A shows the distributions of GHGE by group at baseline according to self-assigned dietary type. People following a vegan diet showed the lowest mean GHGE, and this figure was highest for consumers of mixed diets. No differences in baseline GHGE were discernible between groups. At follow-up, distributions did not change (Fig. 3B).

Figures 2C-D and 3C-D present the distributions of each outcome according to the frequency of consumed cafeteria meals. Neither mean PHDI nor mean GHGE differed by the frequency of consumed cafeteria meals at baseline between intervention groups. At follow-up, the distribution of PHDI remained stable among infrequent consumers (1–5 times per week, *n* = 45), while the group of frequent consumers (≥ 6 times per week, *n* = 1) showed increased PHDI. This was not observed for GHGE. There were no differences between groups. PHDI correlated inversely with GHGE at baseline (*r* = -0·53; *p* < 0·0001) and at follow-up (*r* = -0·49; *p* < 0·0001).

Among individuals in the intervention group who consumed the vegan menu 3 times per week, the mean increase in PHDI points was greatest (*n* = 21, + 1·76 points), followed by individuals with consumption 4–5 times per week (*n* = 8, + 1·63 points), 2 times per week (*n* = 21, + 0·52 points), 1 time per week (*n *= 29, -0·24 points), and never (*n *= 12, -0·33 points). There was no correlation with GHGE.

### Intervention effects on the EAT-Lancet planetary health diet index and GHGE

Table 2 shows the intervention effects from DID analyses for PHDI and GHGE. After the intervention, mean PHDI increased in the intervention group by 0·55 score points and decreased in the control group by -0·06 score points. In the crude Model 1, this translated into a DID of 0·62 (95% CI: -0·35, 1·58; *p* = 0·21). In the fully adjusted Model 3, this effect attenuated to DID = 0·54 (95% CI: -0·51, 1·59; *p* = 0·31). Regarding mean GHGE, we observed an increase in groups from baseline to follow-up (Table 2). Still, the increase was smaller in the intervention as compared to the control group, translating into an overall DID in the crude Model 1 of -0·03 kg CO_2_eq./day (95% CI: -0·16, 0·10; *p* = 0·64). This effect estimate strengthened in Model 3 to -0·07 kg CO_2_eq./day (95% CI: -0·22, 0·08; *p* = 0·34).

**Table 2:** Effects of the intervention on adherence to the Planetary Health Diet Index and on food-related greenhouse gas emissions from the difference-in-differences analysis among 190 hospital employees

Outcomes	Model 1	Model 2	Model 3	Model 4
**Planetary Health Diet Index (0–42 score points)**				
n	190	190	187	190*
**Means at baseline**				
Control (a)	24·74	23·81	22·16	24·72
Intervention (b)	25·10	24·07	22·62	25·11
**Means at follow-up**				
Control (c)	24·68	23·11	22·80	24·72
Intervention (d)	25·65	23·94	23·80	25·68
Difference in intervention e = (d-b)	0·55	-0·13	1·18	0·57
Difference in control f = (c-a)	-0·06	-0·69	0·64	0·01
** Difference-in-differences**	0·62	0·56	0·54	0·56
** 95% confidence interval**	-0·35, 1·58	-0·41, 1·52	-0·51, 1·59	-0·41, 1·54
***p***-value	0·21	0·26	0·31	0·25
**Greenhouse gas emissions (kg CO**_**2**_**e/day)**				
n	180		177	180*
**Means at baseline**		-		
Control (a)	3·32	-	3·59	3·32
Intervention (b)	3·29	-	3·60	3·29
**Means at follow-up**		-		
Control (c)	3·37	-	3·58	3·37
Intervention (d)	3·31	-	3·52	3·31
Difference in intervention e = (d-b)	0·02	-	-0·08	0·01
Difference in control f = (c-a)	0·05	-	-0·01	0·05
** Difference-in-differences**	-0·03	-	-0·07	-0·03
** 95% confidence interval**	-0·16, 0·10	-	-0·22, 0·08	-0·16, 0·10
***p***-value	0·64	-	0·34	0·64

Model 1: Unadjusted

Model 2: Adjusted for educational attainment

Model 3: Adjusted for educational attainment, number of children in the household, occupation

Model 4: weighted by propensity score

^*^Observations with missing covariates were imputed with the mode·

In the examination of individual food groups legumes exhibited significant mean increases in PHDI points (+ 0·48 points), grams (+ 15 g), and GHGE (+ 0·04 kgCO2eq.). We also observed a significant reduction in potato consumption in grams, resulting in a significant increase in PHDI points. Within the dairy category, there was a significant decrease in score points. However, there was no significant increase in grams. Lastly, there were no significant changes in meat categories. In the analyses conducted for each outcome within the male and female participant groups, females showed increasing trends while males showed no trend. We did not observe any significant effects.

### Sensitivity analyses

The propensity-score-weighted DID analysis (Model 4) provided similar results to those of Model 3 (Table 2). The random split yielded a DID estimate for PHDI of 0·62 (95% CI: -0·37, 1·61; *p* = 0·22) and for GHGE of -0·05 (95% CI: -0·18, 0·09; *p* = 0·49). Randomization inference showed that 20% of group re-samplings for PHDI (95%: CI 0·19, 0·21) and 64% of re-samplings for GHGE (95%: CI 0·62, 0·65) showed stronger effects than those measured in our experiment. The ITT analysis assigned participants to groups according to the cafeteria where they completed the baseline questionnaire. For PHDI, the corresponding DID estimate was 0·62 score points (95% CI: -0·35; 1·58 *p* = 0·19) and for GHGE the DID estimate was -0·04 kg CO_2_eq./day (95% CI: -0·18, 0·09; *p* = 0·52). When restricting the analysis to the subgroup who comply with intervention assignment (86%), the LATE for PHDI was 0·71 and for GHGE was -0·05.

## Discussion

In this quasi-experimental study, we introduced vegan menus and educational material on sustainable diets in a cafeteria of a large German hospital over a period of three months. We compared the changes in adherence to the EAT-Lancet PHDI and in GHGE between hospital employees consuming their meals in the intervention and the control cafeterias. Individuals with self-assigned plant-based dietary practices showed higher PHDI score points and lower GHGE than those consuming mixed diets. While the intervention effects using a DID analysis were not significant, the PHDI tended to increase stronger in the intervention group than in the control group. A similar trend was seen for GHGE. Individuals consuming the vegan menu more frequently showed greater, but not significant, increases in PHDI than those never or rarely consuming the vegan menu.

### Distributions of PHDI and GHGE

The mean PHDI of 25 points in this study was about 7 points higher than the mean PHDI in a population-based cohort study with 43–73 year-olds in Sweden; which applied the same approach for PHDI construction [21]. The mean GHGE was 3·3 kg CO2eq./d, comparable to Greenpeace’s estimation for consumers of meat 3–4 times a week [29]. However, in order to stay within planetary boundaries, our participants would have to achieve full points in all PHDI red meat and dairy categories and halve their GHGE [6].

The correlation between PHDI and GHGE in our study is imperfect; participants with high GHGE can attain high score points on the PHDI. This is because both outcomes operationalize different aspects of sustainability. PHDI combines healthfulness with adherence to six planetary boundaries in all relevant food categories, while GHGE characterize only one of those boundaries. The PHDI scoring system rewards high intakes of recommended foods and punishes high intakes of unrecommended foods. Further, the system is based on discrete steps from 0 to 3 points, and only accommodates changes within defined cut-offs for each food group. Reducing beef consumption from three times to once a week does not increase the score, as a points change is only awarded for consuming beef less than once a week. GHGE, on the other hand, is a continuous variable and is very sensitive to dietary changes regarding food groups with high GHGE, emphasizing the effects of diet on climate change.

### Intervention effects on PHDI and GHGE

In this sample of 190 hospital employees, we saw trends for improved adherence to the PHDI and reductions in GHGE after the 3-months intervention period, but no significant effects. Descriptive analyses showed a slightly higher increase in PHDI among participants with self-assigned vegetarian diet than individuals on vegan, pescatarian, flexitarian, and mixed diets. Participants with high PHDI at baseline might have had lower capacity to improve than individuals with low baseline values.

The significant increase in consumption of legumes suggests beneficial effects attributable to our intervention. Since there were no significant differences in the consumption of dairy and meat products, we infer that participants may have compensated for reduced consumption in these categories at home or that reported intakes for these categories were imprecise. This may be due to the detailed breakdown of individual components within dairy and meat categories (dairy: 10, meat: 13) compared to the legume category (4 components), making it easier for participants to lose track of total intake. Ultimately, our GHGE calculation highlighted that an increase in legume consumption without a concurrent reduction in meat and dairy production does not result in decreased emissions.

The study sample size was small as it was not calculated for the outcomes of this paper and the proportion of individuals lost to follow-up was large (72%). Even though the numbers were similar between groups, arguing against selection bias, we run into the possibility of type II error with insufficient power to detect an effect.

Furthermore, previous facility-based diet interventions (FBDI) have had longer durations (6 months to 2 years) or included individual counselling [18]. We did not consider counselling to be an appropriate means for the present intervention programme due to its high demand for human resources. Nevertheless, it appears plausible that participants with high mitigation capacity, namely consumers of vegetarian, flexitarian, and mixed diets will develop stronger PHDI adherence over a longer intervention period. In our intervention group, 18–33% of flexitarians and mixed diet consumers felt motivated by the cafeteria menu to adopt more sustainable dietary practices at home. However, according to the transtheoretical model multiple months to years are required to transition from the stage of contemplation into the stage of action [11].

Information materials and practical tips, as provided in the second component of our intervention, are valuable resources for promoting the transition to action because willingness and capacity to change are associated with knowledge [14]. To transfer to more sustainable diets, consumers need to know that the percentages of animal and plant-based foods in a diet are the most important determinants of sustainable diets. In particular, due to the short duration of our intervention, our educational component did not unfold its full potential. Future studies with longer durations should invest in a more strategic implementation of this component.

Finally, we speculate that the intervention programme was not fundamental enough. In fact, we still offered meat-and-fish menus in the intervention cafeteria. Consequently, participants in the intervention group may not have been subjected to large dietary changes. The effect of the intervention in the group who never consumed the vegan menu was equivalent to control conditions. Indeed, we found higher increases in mean PHDI at higher consumption levels of the vegan menu. To maximise the intervention effects, future studies should concentrate on actively engaging participants who do not initially choose vegan options.

### Strengths and limitations

To date, intervention studies targeting vegan or sustainable consumption in a collective meal context have been scarce, often lack a robust study design, have focused on labelling, prompting, and education [30], and have not increased the availability of sustainable menus. While a recent study reported a 45% increase in consumption of vegetarian dishes after a FBDI for more plant-based diets, there have been no studies reporting effects on individual diets using a priori indices [31]. Also, previous studies lack a common definition of sustainable diets and measure diverse outcomes [32]. This is the first report on the effects of an FBDI on the sustainability of consumers’ diets measured by the PHDI and GHGE.

Using semi-quantitative FFQ data allowed us to rank participants according to their adherence to an a priori index for sustainable diets. Of the currently available diet indices, the Stubbendorff index shows the best performance when estimating health and environmental impacts of diets [33]. We acknowledge that self-reported dietary data from FFQs can be subject to recall bias and underreporting. In addition, this FFQ has not been validated yet. Still, this does not affect the results of our DID analysis, given that all participants carry the same measurement error.

Food choices are influenced by many different factors in addition to the food environment [8]. We have controlled for confounders, observed and unobserved, by means of the DID analysis. Yet, in contrast to randomized controlled trials, DID analyses rely on the common trends assumption [25]. While we could not statistically validate this assumption in our study, conceptual arguments, similar distributions of covariates and between-group distributions of the outcome variables at baseline favour this assumption.

Lastly, there was the possibility of spill-over effects through switching between cafeterias and exchanging information between employees. This might have diluted the actual intervention effects, as could be seen in ITT and LATE. Also, ITT analysis and LATE provided stronger estimates for outcomes. This could be due to a high proportion of those participants already consuming sustainable diets switching to the intervention group.

### Research prospects

Future research in this area should focus on the sustained effects of FBDIs on both health and environmental parameters. When performing short-term FBDIs, we propose limiting research to an accurate estimation of changes in food categories with high environmental impact. We believe that the PHDI is only a sensitive evaluation tool for evaluating FBDIs affecting all daily meals and with long durations because quite radical diet changes in multiple food categories are necessary to substantially increase PHDI. A separate analysis of changes in high-impact food categories is indispensable. To increase the accuracy of food reporting, we propose interviewer administered FFQs and a re-evaluation question on the total intake of each food category at the end of questioning.

While the promotion of plant-based and sustainable diets is an emerging field, it can draw on long-standing experiences from related fields, such as weight-loss and healthy diet interventions [30]. Future studies can use our intervention design as a starting point. However, to animate consumers not initially adopting sustainable food options, interventions should have longer durations and be progressively intensified. This could be accomplished by limiting unsustainable food options, increasing vegan options for all meals and vigorously escalating the educational component. Future studies could also make further investigations into differences in effects depending on gender. Including multiple study sites could provide a larger sample size and greater diversity, enhancing the generalizability of findings.

## Conclusion

This study contributes to bridging the evidence gap on effective strategies to promote sustainable diets and may serve as a starting point for similar contexts. The findings of this study reflect underlying mechanisms of the transtheoretical model and observations from previous studies, strengthening the hypothesis that facility-based approaches can be applied for the promotion of sustainable diets. We encourage future research on the most effective strategies to change global eating patterns. In conclusion, Antonio Guterres’ climate change invocation: “If we don’t act now, this century will be one of humanity’s last. We can build a safer, fairer, more resilient world. But we need to act quickly [34].”

### Supplementary Information

Supplementary Material 1.

## Data Availability

The study protocol and procedures are accessible via the German Clinical Trial Register (DRKS) website under the registration number: DRKS00032620 and on our institutional website: https://www.klinikum.uni-heidelberg.de/klimaschutz-in-kliniken-durch-optimierung-der-lieferketten-kliol. Anonymized participants' data are available upon reasonable request from the principal investigators and for research purposes only. The principal investigators will review an analysis proposal and examine its compliance with the scientific goals of the KLIOL project.
