# Supplementary material for: Effects of a cafeteria-based sustainable diet intervention on the adherence to the EAT-Lancet planetary health diet and greenhouse gas emissions of consumers: a quasi-experimental study at a large German hospital
Source: Nutr J. 2024 Jul 18;23:80. doi: 10.1186/s12937-024-00981-x (PMC11256364; doi:10.1186/s12937-024-00981-x)
Supplement: Supplementary file 1 — Supplementary Material 1. [file 12937_2024_981_MOESM1_ESM.docx]

**Supplementary material**

**Table of contents**

|  |  |  |
| --- | --- | --- |
| Supplementary text 1 | Intervention Design.……………………….………………………………..…….. | 3 |
| Supplementary text 2 | Self-assigned diet patterns……………………………………………...………… | 4 |
| Supplementary text 3 | Dietary Assessment and Construction of Outcomes …………………………….. | 4 |
| Supplementary table 1 | Food components included in the EAT-Lancet Planetary Health Diet Index modified after Stubbendorff et al. (Target intake, calculation and scoring)..…………………………………………………………………………... | 5 |
| Supplementary text 4 | Sample Size Calculation …………………………………………………………. | 6 |
| Supplementary text 5 | Imputation and Exclusion………………………………………………………… | 6 |
| Supplementary table 2 | Individual analysis of food components by PHDI points, grams and GHGE…….. | 7 |
| Supplementary figure 1 | Standardised mean differences in baseline characteristics…………..…………… | 8 |
| References for supplementary material...…………………………….……………….………..…………… | | 9 |

**Supplementary Text 1.** Intervention Design and Menu Plan for One Week

The vegan menu was offered in the intervention cafeteria together with the vegetarian menu and a meat-and-fish-menu from the original standard menu from Mondays to Fridays. It was developed by the study team in consultation with the cooks of the central kitchen, who prepared the menu. The vegan menu was designed to be compatible with the Giessen vegan food pyramid,^1^ the EAT-Lancet Planetary Health Diet,^2^ the Quality Standards for Food Supply in hospitals by the German Society for Nutrition,^3^ and the dietary guidelines of the hospital. It included meals with varying plant-based protein sources e.g. legumes (lentils, beans, soy, tofu, (chick)peas and processed meat substitutes) and whole grains as main components. The proportion of nuts and vegetables was higher than in the standard menu and convenience products were reduced to a minimum. Vegetables were largely delivered daily by a vegetable merchant from local farmers in summer and from European countries in winter. Average GHGE factors in kg CO2eq./meal based on LCAs were calculated for the different menus before project implementation (meat-and-fish =3,25/3,6, vegetarian=0,85, vegan=0,42) and reported in a separate study (Harrison L, unpublished). The vegan menu was placed as the first menu line on the menu and at the beginning of the serving line on entering the cafeteria. All three menu lines received new creative names that deliberately did not including the words “vegan” or “vegetarian”, to avoid adverse reactions to these words.^4^ However, cafeteria staff described the menu as vegan in practice on site.^5^ All dishes in the vegan menu received descriptive titles and the menu line received the name “Fit in the Clinic”, as this was the name of the program of the corporate health management at the hospital.^5^ Each meal received a vegan or vegetarian pictogram or a pictogram of the main animal component. Advertisement before the start of the intervention used the slogan: “Tasty, sustainable, healthy”. All menu lines were sold for the same price. We decided to include educational material for the following reasons: Interventions that rely on fast and intuitive thinking, e.g. presenting sustainable food options at the beginning of the menu line, are more effective than those that rely on slow and cognitively demanding processes,^6^ e.g distributing educational pamphlets.^7^ However, the goal-directed model of behaviour change presents the theory that consumers can only consciously decide to change their diet to a more sustainable one if they perceive a discrepancy between the current state of their diet and sustainable diets. Furthermore, the (re-)evaluation of the effectiveness of selected measures to achieve a health behaviour goal is necessary.^8^ Consumers may believe they are making the greatest possible change to reach sustainable diets when they are eating organically and locally, not knowing that restricting meat is a far more effective strategy.

The performed Intervention in the context of the COM-B Model adapted from Michie et al.:


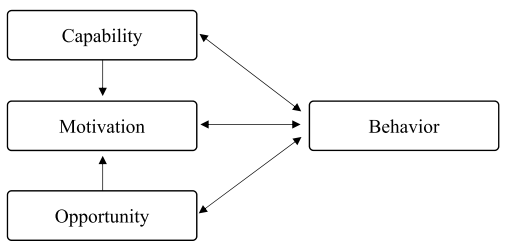

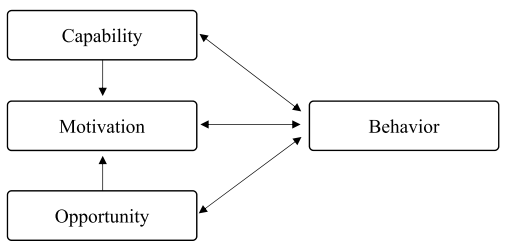


**Psychological capability** through flyers with sustainable diet information

**Physical capability** through an online recipe book to increase cooking skills

**Social opportunity** through positive social norms around sustainable food in hospital with peers and colleagues

**Physical opportunity** through availability in cafeterias

**Reflective motivation** through increasing capability leading to increase in beliefs about capability regarding diet goals

**Automatic motivation** through product positioning in serving line and descriptive meal titles

**Capability**

**Motivation**

**Opportunity**

**Behaviour**

**Supplementary Text 2.** Self-assigned diet patterns

Prior to the intervention, participants were asked to state their diet patterns from the following range:

| Mixed diet | meat or fish more than 3 times a week |
| --- | --- |
| Flexitarian | meat up to two times a week |
| Pescatarian | Mixed diet excluding meat |
| Vegetarian | No meat or fish |
| Vegan | 100% plant-based, no animal products, excluding meat, fish, eggs and dairy |

#### **Supplementary Text 3.** Diet Assessment and Calculation of Outcomes

One food-item (“asparagus (in summer)”) was excluded from all calculations post-study because the study took place in winter. In conclusion, 115 food items were included for the analysis. The average total food intake (g/day) was summarized from the FFQ. Portion sizes were either annotated in the FFQ or calculated on the basis of standard German portion sizes. Total intakes of protein, fat, and carbohydrates in grams were calculated using the national food composition database (BLS 3.01), developed by the Max Rubner-Institute as a standard instrument for the evaluation of nutrition studies in Germany.^9^ Daily energy intake was calculated using the intake of these three macronutrients and standardised to 2500kcal/person/day and g/day of all food items were adjusted proportionally to match the energy intake.

#### EAT-Lancet Planetary Health Diet Index (PHDI)

The PHDI assesses the level of adherence of participants to the EAT-Lancet Planetary Health diet. The score of the index was composed by summing the intake in g/day of all food items from the FFQ into 14 food categories. Based on the recommendations from the EAT-Lancet diet, food components were classified as either “emphasized” or “limited foods”. “Emphasized food components were vegetables, fruits, unsaturated oils, legumes, whole grains, nuts, and fish”^10^. Limited food components were beef and lamb, pork, poultry, eggs, dairy, potatoes, and added sugar. Each food category was assigned points with a possible range of 0–3 points for each component; 0 points for low intake of emphasized foods and 3 points for high intake of emphasized foods and vice versa for limited foods. The final score ranges from 0 (nonadherence) to 42 points (perfect adherence to the EAT-Lancet diet = 14 × 3 points). Dairy, eggs and added sugars from cakes or cookies were also taken into account. Most food categories were calculated almost equal to Stubbendorff et al. with some minor changes due to lacking or different information in food composition tables or FFQs. Histograms for distributions of points for all food categories were computed for the whole study sample at baseline for comparison with previous studies.

*Greenhouse gas emissions*

Greenhouse gas emissions per person per day were calculated by multiplying each food item of the Food-Frequency Questionnaire by factors for CO2-equivalents (CO2eq.) per kg.
These factors were extracted from a comprehensive study conducted by the Institute for Energy and Environment Research (Ifeu) regarding the ecological footprint of German food products.^11^ Specifically, life cycle assessments (LCA) from cradle to supermarket shelf were performed using the methodological framework of ISO standards 14040 and 14044 using an attributive accounting method.^12,13^ In line with ISO 14067 on the carbon footprint of products,^14^ they accounted for all GHGE using conversion factors for CO_2_e, including carbon dioxide (CO_2_), methane (CH_4_), and nitrous oxide (N_2_O).^15^ Land use changes and associated GHGE, in particular those resulting from deforestation for agricultural land use, were taken into account by an attributive land use change approach.^16^ If specific food items were not included in the Ifeu inventory, they received the factor of a similar food item or the average of the food group.

Supplementary Table 1. Food components included in the EAT-Lancet Planetary Health Diet Index according to Stubbendorff et al.^10^ (Target intake ^2^, calculation and scoring).

| **Food component & *Target intake in g**** | **Calculation of food component** | **3 points** | **2 points** | **1 point** | **0 points** |
| --- | --- | --- | --- | --- | --- |
| **Emphasised intake** | | | | | |
| Whole grains  232 | Fiber-rich breakfast cereals (≥10% fiber), rolled oats, fiber-rich soft bread (>4.5% fiber), fiber-rich crispbread (≥10% fiber), fiber-rich rusks >10% fiber. We multiplied the intake for rice and pasta with a factor for the individual percentage of whole grain pasta and rice, derived from a question on the frequency of consumption of whole grain pasta and rice. | >232 | 116–232 | 58–116 | <58 |
| Vegetables  *300*  *(200-600)* | All vegetables except legumes | >300 | 200-300 | 100-200 | <100 |
| Fruits  *200*  *(100–300)* | Fruits and berries | >200 | 100–200 | 50–100 | <50 |
| Fish  *28 (0–100)* | Fatty fish, lean fish, fish products, shellfish | >28 | 14–28 | 7–14 | <7 |
| Legumes  *75 (0–150)* | Dry beans, lentils, peas, soy. Targets and index refer to raw weight. Peas, lentils, beans, tofu, soy containing meat replacement products. | >75 | 37.5–75 | 18.75–37.5 | <18.75 |
| Nuts  *50 (0–100)* | Peanuts or tree nuts. All nuts and seeds including peanuts, nut mixes such as nut paste and chocolate cream. | >50 | 25–50 | 12.5–25 | <12.5 |
| Unsaturated oils  *40 (20–80)* | All plant oils and plant margarines. We added 28g (2 tablespoons) of fat to each diet. This was multiplied by a factor for the percentage of unsaturated fats, derived from a question on choices of oils and fats. | >40 | 20–40 | 10–20 | <10 |
| **Limited intake** | | | | | |
| Dairy  *250 (0–500)* | Whole milk or derivative equivalents. Regular milk, low-fat milk, yoghurt and other fermented milk products, hard cheese, soft cheese, cream, butter, butter-based spreads. In the EAT-Lancet diet all dairy foods are expressed as of milk equivalents. The milk equivalents we used are based on the approach used by Stockholm Resilience Centre, based on ‘total solids’ and intakes of different dairy products were consequently multiplied with the following factors; whole milk 1.0, Cheese 5.0, cream 2.7 and butter 6.5. Furthermore, we included dairy from recipes (e.g., cakes) and multiplied it by the factor derived from a question on the frequency of consumption of dairy in sweet foods. | <250 | 250–500 | 500–1000 | >1000 |
| Beef and lamb  *7 (0–14)* | Beef, lamb, minced meat with pork and lamb, processed meats with beef and lamb including sausages. | <7 | 7–14 | 14–28 | >28 |
| Pork  *7 (0–14)* | Pork, minced meat of pork, processed meats with pork including ham, bacon, and sausages. | <7 | 7–14 | 14–28 | >28 |
| Chicken  *29 (0–58)* | Chicken and other poultry. | <29 | 29–58 | 58–116 | >116 |
| Eggs  *13 (0–25)* | Eggs e.g. boiled, fried. We included egg from recipes (e.g. cakes) and multiplied it by the factor derived from a question on the frequency of consumption of egg in sweet foods. | <13 | 13–25 | 25–50 | >50 |
| Potatoes  *50 (0–100)* | Boiled potatoes, fried potatoes, potatoes included in dishes such as potato salad. | <50 | 50–100 | 100–200 | >200 |
| Added sugar  *31 (0–31)* | Calculation of added sugar was performed by summing the amount of added sugars in all food items. Information on added sugars was obtained from openfoodfacts.org ^17^ using the nutritional information of a random exemplary product for each food item or using exemplary recipes for food items like cakes or cookies. | <31 | 31–62 | 62–124 | >124 |
| All plant drinks were included in respective categories (whole grains, fruits, nuts, legumes) and multiplied by 0.1 as they consist of 10% plant and 90% water. Plant-based alternatives were included in respective categories depending on the main plant-basis specified in FFQ. | | | | | |

*Target intake for the EAT-Lancet Planetary Health Diet is reported in possible ranges of macronutrient intakes for food groups. Depending on the level of intake for one food group, the possible intake for another food group may increase or decrease within the possible range to ensure healthy nutrition while staying within boundaries for planetary health e.g., 58g/day chicken intake, no beef intake.

**Supplementary Text 4.** Sample Size Calculation

The sample size calculation was performed for the primary outcome mental well-being, assessed by the Warwick-Edinburgh-Mental-Wellbeing-Scale (WEMWBS). Based on previous intervention trials that employed dietary modifications to determine effects on wellbeing,^18-20^ we calculated the sample size for paired t-tests (before- and-after studies).^21^ We assumed an effect size ranging between 0.18 and 0.30 WEMWBS score points (α = 0.05, 1−β= 0.8), which required a sample size of 90 to 245 participants. To account for potential loss to follow-up, we aimed at enrolling 2x250 individuals.

**Supplementary Text 5.** Imputation and Exclusion

Observations with only 1 missing food-item per category were imputed with the sample mean. Some observations had complete missing food categories, e.g. all plant-based meat and dairy alternatives. This was especially the case in follow-up observations. As it was possible to exclude entire food categories at the beginning of the questionnaire, the fact, that some whole categories were missing could have been due to errors in LimeSurvey. If multiple food-items in one or two categories or in multiple related categories (e.g. all plant alternatives) were missing, we supplemented the missing values with values from baseline or follow-up (n=16). Observations with a large amount of supplemented values were checked for similar energy intake after supplementation. This rendered differences up to around 500 kcal. 9 observations with many unrelated missing food categories were excluded. 17 observations with very high or low and extremely variable energy intake were excluded. For low energy intake we chose a cut-off of 800kcal. Diets below 800kcal can be defined as very-low-calorie ketogenic diets.^22^ Participants with energy intakes under this cut-off were rated as following a strict dietary regimen, which could restrict food choices and was a reason for exclusion. Furthermore, we excluded diets with energy intakes higher than 3500kcal for women (except if this high intake was the same for both time points) and for men if there was extreme variability in energy intake over both time points, as suggested by Willett. ^23^

**Supplementary Text 6**. Food Frequency Questionnaire Design

The study team developed a food frequency questionnaire with 116 items based on the German food-based dietary guidelines in order to capture plant-based diet patterns.^24^ Questions pertained to the following categories: bread, vegetables, fruits, fish, legumes, nuts, fats, dairy, plant-based drinks, other plant-based dairy substitutes (e.g. plant-based yoghurt), meat, meat substitutes (e.g. seitan), eggs, side-dishes (e.g. noodles, rice, potatoes), sweets, drinks, spreads (e.g. butter, jam).

Supplementary Table 2. Mean values and difference in different outcomes of food components by PHDI points, grams and GHGE

|  | **Points per PHDI category (0=lowest to 3=highest)** | | | | | | | **Grams per PHDI category*** | | | | | | | **GHGE in kgCO2eq. from questionnaire categories** | | | | | | | |
| --- | --- | --- | --- | --- | --- | --- | --- | --- | --- | --- | --- | --- | --- | --- | --- | --- | --- | --- | --- | --- | --- | --- |
|  | **Baseline** | | **Follow-up** | |  |  |  | **Baseline** | | **Follow-up** | |  |  |  |  | **Baseline** | | **Follow-up** | |  |  |  |
|  | **CG** | **IG** | **CG** | **IG** | **DID** | **p-value** |  | **CG** | **IG** | **CG** | **IG** | **DID** | **p-value** |  |  | **CG** | **IG** | **CG** | **IG** | **DID** | **p-value** |  |
| **Emphasised intake** | | | | | | | | | | | | | | | | | | | | | | |
| **Whole grains** | 1.68 | 1.82 | 1.71 | 1.96 | **0.11** | 0.35 |  | 150 | 160 | 168 | 181 | **3** | 0.74 |  | **Bread** | 0.06 | 0.06 | 0.06 | 0.06 | **0.00** | 0.76 |  |
| **Vegetables** | 2.64 | 2.78 | 2.68 | 2.82 | **-0.01** | 0.93 |  | 504 | 516 | 539 | 551 | **-1** | 0.97 |  | **Vegetables** | 0.20 | 0.20 | 0.21 | 0.21 | **-0.01** | 0.69 |  |
| **Fruits** | 2.59 | 2.60 | 2.55 | 2.46 | **-0.10** | 0.30 |  | 350 | 296 | 331 | 291 | **14** | 0.62 |  | **Fruits** | 0.19 | 0.19 | 0.20 | 0.18 | **-0.02** | 0.38 |  |
| **Fish** | 1.87 | 1.86 | 1.90 | 1.90 | **0.01** | 0.93 |  | 30 | 34 | 33 | 32 | **-6** | 0.22 |  | **Fisch** | 0.18 | 0.21 | 0.19 | 0.19 | **-0.03** | 0.26 |  |
| **Legumes** | 1.23 | 1.41 | 1.19 | 1.85 | **0.48** | 0.0003 |  | 50 | 48 | 53 | 67 | **15** | 0.02 |  | **Legumes** | 0.10 | 0.09 | 0.10 | 0.12 | **0.04** | 0.004 |  |
| **Nuts** | 1.17 | 1.14 | 1.36 | 1.09 | **-0.24** | 0.10 |  | 27 | 31 | 28 | 27 | **-5** | 0.22 |  | **Nuts** | 0.02 | 0.02 | 0.02 | 0.02 | **0.00** | 0.15 |  |
| **Unsaturated Oils** | 1.82 | 1.89 | 1.81 | 1.82 | **-0.07** | 0.35 |  | 27 | 27 | 27 | 27 | **0** | 0.95 |  | **Fats** | 0.12 | 0.11 | 0.11 | 0.11 | **0.01** | 0.20 |  |
| **Limited intake** | | | | | | | | | | | | | | | | | | | | | | |
| **Dairy** | 1.21 | 1.52 | 1.28 | 1.28 | **-0.30** | 0.01 |  | 711 | 677 | 599 | 623 | **58** | 0.21 |  | **Dairy** | 0.81 | 0.79 | 0.66 | 0.73 | **0.09** | 0.14 |  |
|  |  |  |  |  |  |  |  |  |  |  |  |  |  |  | **Plant drinks** | 0.03 | 0.04 | 0.03 | 0.03 | **-0.01** | 0.19 |  |
|  |  |  |  |  |  |  |  |  |  |  |  |  |  |  | **Other dairy substitutes** | 0.01 | 0.01 | 0.01 | 0.01 | **0.00** | 0.37 |  |
| **Beef and Lamb** | 1.26 | 1.32 | 1.28 | 1.46 | **0.12** | 0.39 |  | 29 | 28 | 28 | 33 | **6** | 0.34 |  | **Meat** | 0.57 | 0.60 | 0.58 | 0.55 | **-0.07** | 0.20 |  |
| **Pork** | 1.33 | 1.17 | 1.38 | 1.38 | **0.16** | 0.23 |  | 30 | 30 | 34 | 33 | **-1** | 0.84 |  | **Meat substitutes** | 0.04 | 0.04 | 0.04 | 0.04 | **0.00** | 0.89 |  |
| **Poultry** | 2.64 | 2.54 | 2.60 | 2.60 | **0.10** | 0.28 |  | 21 | 23 | 25 | 23 | **-4** | 0.20 |  |  |  |  |  |  |  |  |  |
| **Eggs** | 1.52 | 1.46 | 1.35 | 1.29 | **0.01** | 0.94 |  | 33 | 36 | 32 | 35 | **-1** | 0.65 |  | **Eggs** | 0.08 | 0.10 | 0.09 | 0.10 | **-0.01** | 0.28 |  |
| **Potatoes** | 1.83 | 1.60 | 1.61 | 1.73 | **0.34** | 0.003 |  | 92 | 107 | 111 | 106 | **-20** | 0.04 |  | **Side-dishes** | 0.26 | 0.29 | 0.31 | 0.35 | **0.01** | 0.71 |  |
| **Added sugars** | 1.95 | 1.99 | 1.99 | 2.03 | **0.00** | 0.98 |  | 51 | 48 | 50 | 47 | **0** | 0.94 |  | **Sweets** | 0.16 | 0.16 | 0.16 | 0.13 | **-0.02** | 0.13 |  |
|  |  |  |  |  |  |  |  |  |  |  |  |  |  |  | **Drinks** | 0.38 | 0.33 | 0.38 | 0.34 | **0.00** | 0.90 |  |
|  |  |  |  |  |  |  |  |  |  |  |  |  |  |  | **Spreads** | 0.12 | 0.12 | 0.13 | 0.13 | **0.00** | 0.82 |  |

* Grams calculated as described in Supplemental table 1

**Supplementary Figure 1.** Standardised mean differences in baseline characteristics ^25^

*LTR= Long-term relationship
 MPA= Moderate ph1ysical activity

**References for Supplementary Material:**

1. Weder S. Schaefer C. Keller M. Die Gießener vegane Lebensmittelpyramide. *Ernahrungs Umschau* 2018; **65**(8): M422-M31.

2. Willett W. Rockstrom J. Loken B. al e. Food in the Anthropocene: the EAT-Lancet Commission on healthy diets from sustainable food systems. *Lancet* 2019; **393**(10170): 447-92.

3. DGE-Qualitätsstandard für die Verpflegung in Kliniken. In: e.V DGfE. editor. 1. 1. korrigierter Nachdruck ed; 2022.

4. Cole RE. Bukhari AS. Champagne CM. McGraw SM. Hatch AM. Montain SJ. Performance Nutrition Dining Facility Intervention Improves Special Operations Soldiers’ Diet Quality and Meal Satisfaction. *Journal of Nutrition Education and Behavior* 2018; **50**(10): 993-1004.

5. Bacon L. Krpan D. (Not) Eating for the environment: The impact of restaurant menu design on vegetarian food choice. *Appetite* 2018; **125**: 190-200.

6. Gordon K. Dynan L. Siegel R. Healthier Choices in School Cafeterias: A Systematic Review of Cafeteria Interventions. *The Journal of Pediatrics* 2018; **203**: 273-9.e2.

7. Prochaska JO. Velicer WF. The Transtheoretical Model of Health Behavior Change. *American Journal of Health Promotion* 1997; **12**(1): 38-48.

8. Vermeir I. Weijters B. De Houwer J. al e. Environmentally Sustainable Food Consumption: A Review and Research Agenda From a Goal-Directed Perspective. *Frontiers in Psychology* 2020; **11**.

9. Bundeslebensmittelschlüssel Was ist der BLS? <https://blsdb.de/bls?background> (accessed 19/08/2023.

10. Stubbendorff A. Sonestedt E. Ramne S. Drake I. Hallström E. Ericson U. Development of an EAT-Lancet index and its relation to mortality in a Swedish population. *The American Journal of Clinical Nutrition* 2022; **115**(3): 705-16.

11. Reinhardt G. Gärtner S. Wagner T. Ökologische Fußabdrücke von Lebensmitteln und Gerichten in Deutschland. In: Heidelberg IfE-uU. editor. Heidelberg; 2020.

12. Iso. ISO - ISO 14040:2006 - Environmental management — Life cycle assessment — Principles and framework. 2006.

13. ISO. ISO 14044:2006 - Environmental management - Life cycle assessment - Requirements and guidelines. . International Organization for Standardization.; 2006b.

14. ISO. ISO 14067:2018 - Greenhouse gases — Carbon footprint of products — Requirements and guidelines for quantification. 2018.

15. IPCC. Climate Change 2013: The Physical Science Basis. Contribution of Working Group I to the Fifth Assessment Report of the Intergovernmental Panel on Climate Change. *Cambridge University Press* 2013.

16. Fehrenbach H. Keller H. Abdalla N. Rettenmaier N. Attributive Landnutzung (aLU) und attributive Landnutzungsänderung (aLUC) Eine neue Methode zur Berücksichtigung von Landnutzung und Landnutzungsänderung in Ökobilanzen. 2020; (2.1 ).

17. Open Food Facts - Freie Datenbank für Lebensmittel. <https://de.openfoodfacts.org/>.

18. Davison J. Stewart-Knox B. Connolly P. Lloyd K. Dunne L. Bunting B. Exploring the association between mental wellbeing. health-related quality of life. family affluence and food choice in adolescents. *Appetite* 2021; **158**.

19. Hayhoe R. Rechel B. Clark AB. Gummerson C. Smith SJL. Welch AA. Cross-sectional associations of schoolchildren’s fruit and vegetable consumption. and meal choices. with their mental well-being: a cross-sectional study. *BMJ Nutrition. Prevention & Health* 2021; **4**(2): e000205-e.

20. Johnson R. Robertson W. Towey M. Stewart-Brown S. Clarke A. Changes over time in mental well-being. fruit and vegetable consumption and physical activity in a community-based lifestyle intervention: a before and after study. *Public health* 2017; **146**: 118-25.

21. Dhand N. Khatkar M. Statulator: An online statistical calculator. Sample Size Calculator for Comparing Two Paired Means. Accessed 20 September 2022 at <http://statulator.com/SampleSize/ss2PM.html>. 2014.

22. Caprio M. Infante M. Moriconi E. al e. Very-low-calorie ketogenic diet (VLCKD) in the management of metabolic diseases: systematic review and consensus statement from the Italian Society of Endocrinology (SIE). *Journal of Endocrinological Investigation* 2019; **42**(11): 1365-86.

23. Willett W. Nutritional Epidemiology: Oxford University Press; 1998.

24. DGE. Vollwertig essen und trinken nach den 10 Regeln der DGE. 10 ed: Deutsche Gesellschaft für Ernährung e.V.; 2017.

25. Warton ME. Parker MM. Oops. I D-I-D It Again! Advanced Difference-in-Differences Models in SAS ®. lexjansen; 2018. p. 25-2018.
